# Validation of the Swedish version of the safe environment for every kid (SEEK) parent screening questionnaire

**DOI:** 10.1186/s12889-023-16792-4

**Published:** 2023-10-12

**Authors:** Maria Engström, Sara Lindqvist, Staffan Janson, Inna Feldman, Howard Dubowitz, Steven Lucas

**Affiliations:** 1 Department of Women’s and Children’s Health, Uppsala University, SE-751 85 Uppsala, Sweden; 2 Division of Public Health Sciences, Department of Health Sciences, Karlstad University, Karlstad, Sweden; 3 Department of Public Health and Caring Sciences, Uppsala University, Uppsala, Sweden; 4 Department of Pediatrics, University of Maryland School of Medicine, Baltimore, MD USA

**Keywords:** Psychosocial risk factors, Child health, Child maltreatment, Prevention, Health promotion, Psychometrics, Validation, Child health services, Evidence-based practice, Women, Men

## Abstract

**Background:**

Psychosocial risk factors in the home may impair children’s health and development and increase the risk of maltreatment. The Safe Environment for Every Kid (SEEK) model was developed to provide pediatric primary care professionals with a structured way to identify common psychosocial problems. The SEEK model includes use of the Parent Screening Questionnaire (SEEK-PSQ) at routine preventive child health visits, discussion with parents about their responses and, when indicated, referral to relevant services. The SEEK-PSQ has not previously been available in Swedish. The aim of the present study was to evaluate the psychometric properties of an adapted Swedish version of the SEEK-PSQ (PSQ-S).

**Methods:**

This study is part of a cluster-randomised controlled trial of SEEK in the Swedish child health services. To validate the PSQ-S, parents (n = 852) with children 0–18 months of age were invited to complete a survey including the PSQ-S as well as evidence-based standardized instruments for the targeted psychosocial risk factors: economic worries, depressive symptoms, parental stress, alcohol misuse and intimate partner violence (IPV). Baseline data from 611 (72%) parents were analysed regarding sensitivity, specificity, positive predictive value (PPV) and negative predictive value (NPV) for each risk factor.

**Results:**

As a whole, the PSQ-S had a sensitivity of 93%, specificity of 52%, PPV of 67% and NPV of 87%. For mothers and fathers combined, sensitivity was 80% for economic worries, 89% for depressive symptoms, 78% for parental stress, 47% for intimate partner violence (IPV) and 70% for alcohol misuse. Specificity was highest for IPV and alcohol misuse (91%) and lowest for depressive symptoms (64%). NPV values were high (81–99%) and PPV values were low to moderate (22–69%) for the targeted problems. Sensitivity was higher for mothers compared to fathers for economic worries, depressive symptoms and IPV. This difference was particularly evident for IPV (52% for mothers, 27% for fathers).

**Conclusion:**

The SEEK-PSQ-S demonstrated good psychometric properties for identifying economic worries, depressive symptoms, parental stress and alcohol misuse but low sensitivity for IPV. The PSQ-S as a whole showed high sensitivity and NPV, indicating that most parents with or without the targeted psychosocial risk factors were correctly identified.

**Trial registration:**

ISRCTN registry, study record 14,429,952 (10.1186/ISRCTN14429952) Registration date 27/05/2020.

**Supplementary Information:**

The online version contains supplementary material available at 10.1186/s12889-023-16792-4.

## Background

The United Nations Convention on the Rights of the Child states that every child has a right to the highest attainable standard of health and a childhood free from violence and neglect [1]. The environment in which the child lives is influenced by many things, and an understanding of the child’s living conditions including identification of risk- and protective factors can help promote their health and development [2]. Psychosocial risk factors in the home environment may impair children’s health and development and increase the risk of child maltreatment (CM) [3, 4]. These risk factors include poverty [5], alcohol or other drug abuse [6], mental illness [7], intimate partner violence (IPV) [8] and major parental stress [9].

CM, defined by the World Health Organization as “the perpetration of physical, sexual and psychological/emotional violence and neglect of infants, children and adolescents aged 0–17 years by parents, caregivers and other authority figures”, is highly prevalent and remains a major public health and social welfare problem [3, 10]. An overview of prevalence studies from 96 countries showed that over half of all children aged 2–17 years had experienced emotional, physical or sexual violence in the past year [11]. In high-income countries, it is estimated that 4–16% of children are physically abused and one in ten are exposed to neglect or psychological abuse every year [3].

The youngest and most vulnerable children are often those most exposed to CM. The risk of maltreatment among children 0–4 years is twice that of children 5–14 years [12]. CM may lead to an array of of physical, psychological and behavior problems in both the short and long term [3, 4, 13], and the risk of health consequences due to abuse increases in a dose-dependent fashion; the more types of abuse a child is exposed to, the greater the risk of poor health outcomes [14]. In addition, the risk factors associated with CM are interrelated and often aggregate in the same families [15]. Despite its high prevalence and negative impact, most of CM goes undetected and is grossly underestimated in official statistics from law enforcement, social welfare and health care [3]. This underscores the need for methods to universally identify children exposed to or at risk of CM.

There is evidence that CM may be prevented through programs that address its causes and risk factors [12, 16–19]. When CM is detected, or when prominent risk factors for CM are identified, evidence-based support to parents provided through the social services or mental health professionals can decrease the risk of future exposure to CM and improve child behavioral outcomes [20, 21]. Using universal screening (e.g. screening all families in a primary care practice) eliminates the stigma of screening selected families and reduces the likelihood of missing at-risk families. Brief tools have been shown to be effective in screening for psychosocial risk factors in primary care [17, 18, 22]. The Safe Environment for Every Kid (SEEK) model helps identify and address psychosocial problems, and aims to strengthen families, support parents and parenting, and thereby promote children’s health, development, wellbeing and safety, and help prevent child abuse and neglect [17, 18].

Evaluations of the the SEEK-model in two randomized controlled trials in the U.S. showed that the professionals who used the model felt more secure in addressing psychosocial risk factors and did so more often in their practice compared to professionals in the control group [17, 18]. In addition, parents’ use of harsh punishment and the number of reports to child protective services decreased in the intervention group. The U.S. version of the SEEK-PSQ has shown moderately good sensitivity, specificity and predictive values [23–26], but has not been evaluated for internal consistency. Translated versions of the SEEK-PSQ include Spanish, French, Italian, Chinese, Purtugese and Nepali, although these versions have not been validated. A Swedish language version of the PSQ has not previously been available.

The Swedish child health services (CHS) is a primary care-based organization that offers infants and preschool-aged children (0–6 years) regular health visits at dedicated child health centers (CHCs) free of charge [27], and reaches nearly all families [28]. The program is staffed by specialist district or paediatric nurses and general practitioners, with good continuity of care and at least 17 scheduled visits throughout the child’s first six years of life. In contrast to health care in general, the CHS focuses on health promotion and primary prevention through universal and targeted interventions. The CHS thereby plays an important role in providing equitable access to health services and promoting health and development for all children and families [27].

At present, the CHS lacks a systematic and structured approach to identifying common psychosocial risk factors in the home environment that increase the risk of CM. Within the framework of a broader randomized study of the SEEK model in the Swedish CHS, the aim of the present study was to evaluate the psychometric properties of a newly developed Swedish version of the SEEK-PSQ (PSQ-S) compared to standardized lengthier instruments. As previous research has indicated that the performance of some established screening instruments for psychosocial problems differs between genders [29, 30], potential differences between mothers and fathers in this regard were also evaluated in the present investigation.

## Method

### Setting

This study is part of the BarnSäkert (“Child Safe”) project, a longitudinal cluster-randomized controlled trial evaluating the validity, clinical utility and effectiveness of the SEEK model in the Swedish CHS context. The psychometric evaluation presented here is based on baseline data from parents whose children were enrolled in one of the 27 participating CHCs in the county of Dalarna prior to initiation of the randomized trial.

### Development of the PSQ-S

The core components of the SEEK model include: (1) training for pediatric primary care professionals on briefly assessing and initially addressing psychosocial risk factors, and integration of the model into routine child health visits; (2) use of the Parent Screening Questionnaire (SEEK-PSQ); (3) assessment of parents’ responses using elements of Motivational Interviewing; and (4) guidance or referral to healthcare or community resources [14]. The original SEEK model has been adapted to the Swedish CHS through a multi-phase development process.

Translation and adaptation of the original SEEK PSQ followed a process congruent with recommendations from the WHO [31]. First, the PSQ was translated from English to Swedish by the senior author (Steven Lucas), who is bilingual and has extensive experience in social pediatrics. Literal translation was avoided to achieve a culturally sensitive representation of each of the targeted risk factors: child safety issues, economic worries, depressive symptoms, IPV and substance misuse. An expert group including CHS nurses, child psychologists, pediatricians and the creator of the SEEK model (Howard Dubowitz) reviewed the initial Swedish translation. There are a number of societal dissimilarities between the U.S. and Sweden, for example, food insecurity and handgun ownership are relatively uncommon in Sweden and corporal punishment is illegal. The expert group discussed these differences and suggested changes in the wording of some items to better mesh with conditions in Sweden. The expert group also suggested testing two versions of the PSQ, one with three items regarding substance abuse and three items regarding IPV (version A) and one with six items regarding substance abuse and one item regarding IPV (version B).

Six CHS nurses from the expert group piloted the first versions of the Swedish PSQ for three months in their daily practice in a crossover design; three nurses started with version A and three with version B and both groups switched to the other version after six weeks. The expert group then reconvened and reviewed the nurses’ experiences, and a hybrid (version C) was agreed upon, with four items regarding substance abuse and three regarding IPV. Version C was piloted by the same CHS nurses for six weeks. Analysis of the pilot data showed that a large proportion parents reported child safety issues, economic worries, depressive symptoms or parental stress, but very few disclosed substance abuse or IPV. The expert group therefore agreed on a fourth version (version D) which included the AUDIT-C to identify alcohol misuse and two detailed questions to elicit responses regarding psychological IPV and controlling behavior and physical IPV that had previously been used successfully in a national survey of violence exposure among adults in Sweden (Violence and health in Sweden). Version D was piloted for six weeks by the same nurses and showed several-fold higher rates of disclosure for alcohol misuse and IPV compared to the previous versions. This version, called the PSQ-S (PSQ-Swedish version) was then used in the randomized controlled trial of the SEEK model.

**Fig. 1:**
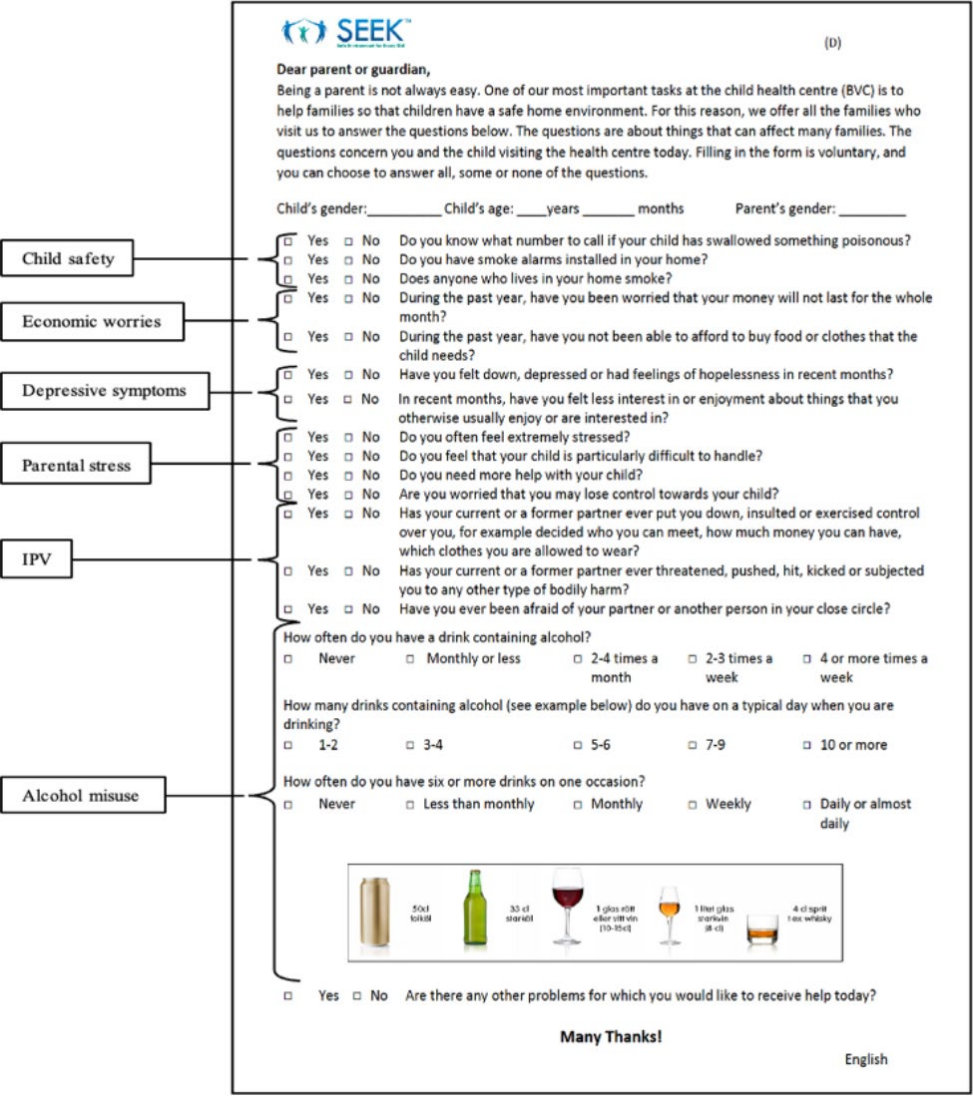
PSQ-S Grouped by risk factors

The final PSQ-S contained 17 questions, with two to four items for each of the six domains (see Fig. 1). Differences compared to the US version of the PSQ used in 2012 are shown in additional file 1 and include a wider time frame for questions regarding depressive symptoms (recent months instead of past month), rephrasing of questions regarding food insecurity and corporal punishment, addition of questions regarding psychological IPV/controlling behavior and physical IPV and replacement of questions regarding alcohol misuse with the AUDIT-C.

The final PSQ-S was translated by a professional translation agency from Swedish to English, Arabic, Somali, Tigrinia, Kurmanji and Dari and back-translated into Swedish by a different professional translation agency. The translated and back-translated PSQ-S forms were reviewed by bilingual, university educated native speakers of each respective language together with the project leaders and corrections were made according to their combined opinions.

The PSQ-S and the US version of PSQ are presented side by side in the supplementary material.

### Structure of the validation questionnair

Data for the longitudinal part of the Child Safe study were gathered using a questionnaire including the PSQ-S and five standardized instruments for the psychometric comparisons. Data from the baseline questionnaire administered to parents before the intervention were used in the present study. Demographic information included the respondent’s age, educational level, occupation, marital status, country of birth of the respondent and his/her parents, and the number of children living in the household.

### Standardized Instruments

#### Economic worries

The Swedish National Public Health Survey, a recurring survey of living conditions by the Public Health Agency of Sweden, contains two items measuring the individual’s financial vulnerability: (1) “If you suddenly found yourself in an unexpected situation where you needed to raise 15 000 SEK (1500 USD) within a week, would you be able to do so?”; (2) “During the past 12 months, have you had difficulties paying your monthly costs for food, rent, bills etc.?”. The questions have shown a clear association to respondents’ socioeconomic status and general state of health [32].

#### Depressive symptoms

The Hospital Anxiety and Depression Scale (HADS) is not a diagnostic test but is well-documented for assessing the presence and severity of symptoms regarding anxiety disorders and depression and has been applied in health care settings as well as in the general population [33–35]. The HADS consists of seven items each for anxiety and depression measured on a four point (0–3) Likert scale [34]. Scores for each item are summed to create total scores for depression and anxiety, respectively. Only the depression score was used in the present analyses.

#### Parental stress

The Swedish Parenthood Stress Questionnaire (SPSQ) is a 34-item measure of parental stress among parents with young children and was adapted from the Parenting Stress Index [36]. Response options range from *strongly disagree* to *strongly agree* on a 5-point Likert scale [1–5] with higher scores indicating higher levels of stress [37]. Scores from the five subscales (*Incompetence*, *Role Restriction*, *Social Isolation*, *Spouse Relationship Problems* and *Health Problems*) are combined to create a total *General Parenting Stress* score which was used in the present analyses.

#### Intimate-partner violence

The Composite Abuse Scale (CAS) is a widely used questionnaire that covers physical, emotional, and sexual abuse in a relationship with a romantic partner and the frequency of such experiences during the last 12 months. The frequency is quantified into never, only once, several times, once per month, once per week and daily. The wording of the 30 items is gender neutral but derives from women´s descriptions of abuse, reports from professionals and court and police reports of IPV. The CAS has not been validated for men [38, 39]. Any positive response regarding physical or emotional violence or fear of a current or previous partner was considered as a positive screen.

#### Substance misuse/abuse

The Alcohol Use Disorders Identification Test (AUDIT) is commonly used in clinical practice to identify harmful patterns of alcohol consumption and dependence. Its 10 questions cover 3 domains: *hazardous alcohol use* (typical quantity and frequency of drinking and heavy drinking), *dependence symptoms* (impaired control over drinking, increased salience of drinking and morning drinking), and *harmful alcohol use* (guilt after drinking, blackouts, alcohol-related injuries, and others concerned about drinking) [40]. The total score from all three domians was used in the present analyses.

Details regarding the content of the PSQ-S and the standardized instruments used for comparison are provided in Table 1. Questions related to child safety were not included in the present study.

**Table 1:** SEEK targeted problems and criteria for positivity in the PSQ-S and corresponding standardized instruments

Dimension	Number of items in the PSQ-S	Criterion for positivity in the PSQ-S	Standardized instrument	Number of items in the standardized instrument	Criterion for positivity in the standardized instrument	Internal consistency (Cronbach’s α)
Economic worries	2	Any positive response	Swedish National Public Health Survey	2	Any positive response	Not available
Depressive symptoms	2	Any positive response	HADS	7	≥ 7 points = possible depression	0.82–0.90
Parental Stress	4	Any positive response	SPSQ	34	≥ 104 points (90th percentile)	0.89
IPV	3	Any positive response	CAS	30	Any positive response	> 0.6
Alcohol misuse	3	≥ 4 points for women ≥ 5 points for men	AUDIT	10	≥ 6 points for women ≥ 8 points for men	0.8
Total:	14			83		

### Sample

The present analysis used a cross-sectional design with a stratified, self-selected sampling procedure. Recruitment of participants was carried out between February 2018 and January 2019. Parents of children 0–18 months of age enrolled in the CHS in Dalarna county were invited to participate. The only inclusion criterion was knowledge of the Swedish language sufficient to understand information about the study and respond to the questions in the survey as assessed by the CHS nurse in her clinical contact with the parent. In connection with regular child health visits at Child Health Centers (CHCs), parents were given general information from the CHS nurse about the Child Safe project and asked if they were interested in participating. Parents who expressed an interest filled in a contact form which was forwarded to the study coordinator who provided oral and written information about the study. Parents who agreed to participate signed an informed consent form which was returned by regular mail to the study coordinator. A total of 852 parents of 704 children from both SEEK and control CHCs consented to participate. For 148 families, two parents were enrolled.

### Data collection and participants

This study included only data from the baseline survey from parents at both SEEK and control CHCs before initiation of the intervention. Questionnaires were distributed and data were collected and managed using Research Electronic Data Capture (REDCap), [41, 42]. The parents were invited to respond to the web-based survey through a link e-mailed to them. A few parents (n = 26) requested a hard copy of the survey that was sent by regular mail. Responses from the paper surveys (n = 19) were added manually before the analyses. Up to four reminders were sent.

The response rate for the baseline survey was 73% (n = 619), 233 parents who had given their informed consent did not respond to the questionnaire despite reminders. Eight web-based surveys were excluded as they were incomplete, giving a final response rate of 72% (n = 611).

The background characteristics of the respondents are presented in Table 2.

**Table 2:** Background characteristics of the respondents

	Mothers	Fathers	Total*
**Respondents** n (%)	500 (82)	111 (18)	611 (100)
**Age in years** mean (SD)	32 (5)	35 (6)	32 (5)
**Education** n (%)	494 (82)	111 (18)	605 (100)
Primary education, 9–10 years	9 (2)	4 (4)	13 (2)
Secondary/high school education, 2–3 years	159 (32)	45 (40)	204 (34)
College level education	326 (66)	62 (56)	388 (64)
**Respondents’ country of birth** n (%)	494 (82)	111 (18)	605 (100)
Sweden	461 (93)	108 (97)	569 (94)
Outside Sweden	33 (7)	3(3)	36 (6)

*n varied slightly between characteristics due to missing data

Chi-square analyses showed no significant differences between genders with respect to educational level (p = 0.10) or country of birth (*p* = 0.12). A higher proportion of participants had a college level education (*p* < 0.001) and fewer were born abroad (p < 0.001) compared to national statistics for women and men 25–44 years of age in Sweden [43]. Eligible participants who opted out were more likely to be men (31%, chi-square; *p* = < 0.001), and slightly younger (mean 31 years) compared to those who did respond (mean 32 years) (*t*-test; *p* = 0.010).

### Data analysis

Pearson’s chi-square was used for dichotomous variables and t-test for continuous variables to analyze potential differences with respect to background factors between genders among participants, between participants and the general population of women and men 25–44 years of age and between respondents and non-responders. Given the use of baseline data, responses from all parents were analysed without regard to randomization status. Sensitivity, specificity, positive predictive value (PPV) and negative predictive value (NPV) were calculated for each targeted problem in the PSQ-S with the corresponding standardized instruments as the reference test. The data in both the PSQ-S and the validated instruments were dichotomized before analysis. Differences in proportions of positive screens between genders and between the PSQ-S and the standardized instruments were analyzed using Pearson’s chi-square, as were differences between genders regarding sensitivity, specificity, NPV and PPV. Internal consistency was analyzed using Cronbach’s α. Exploratory factor analysis applying varimax rotation and a minimum Eigen value of 1.0 was used to evaluate the factor structure of the 14 items of the PSQ-S after excluding questions regarding child safety issues. Factor loading values above 0.3 were considered salient enough to be practically meaningful. The analyses were carried out using SPSS Statistics 28.0 (IBM SPSS Statistics for Macintosh, Version 28.0).

## Results

The descriptive statistics from the PSQ-S and each standard instrument are presented in Table 3. On the PSQ-S, about two thirds of parents reported at least one psychosocial problem compared to about half on any of the standard instruments. For nearly all risk factors, the percentage of parents with a positive screen was significantly higher the PSQ-S compared to the corresponding standard instrument. The exception was IPV, which was significantly higher in the CAS compared to the PSQ-S. The only difference in positivity rates between genders was seen for the full AUDIT questionnaire, where men showed significantly higher rates than women.

**Table 3:** Positivity* on the PSQ-S and the standardized instruments by gender and in total

	Mothers (n = 500)		Fathers (n = 111)		Total (n = 611)	
	%	n (total)	%	n (total)	%	n (total)
**PSQ-S**						
Economic worries	**28**	139 (494)	19	21 (109)	**27**	160 (603)
Depressive symptoms	**43**	212 (494)	**41**	45 (109)	**43**	257 (603)
Parental stress	**31**	147 (480)	**23**	24 (106)	**29**	171 (586)
IPV	**21**	102 (486)	**13**	14 (108)	**20**	116 (594)
Alcohol misuse	**11**	54 (493)	13	14 (109)	**11**	68 (602)
Any positive screen on the PSQ-S	**69**	337 (487)	**64**	68 (107)	**68**	405 (594)
**Standardized instrument for each risk factor**						
Economic worries	**16**	77 (492)	14	15 (111)	**15**	92 (603)
Depressive symptoms	**15**	68 (464)	**12**	12 (104)	**14**	80 (568)
Parental stress	**11**	47 (413)	**8**	8 (90)	**11**	54 (503)
IPV	**29**	133 (454)	**33**	34 (104)	**30**	167(558)
Alcohol misuse	***2***	11 (464)	*9*	9 (104)	**4**	20 (568)
Any problem found on standard instruments	**51**	222 (434)	**52**	51 (98)	**51**	273 (532)

*Significant differences in positivity rates between genders are denoted in italics (chi-square, *p* < 0.05). Significant differences in positivity rates between the PSQ-S and standardized instruments are denoted in bold (chi-square, *p* < 0.05)

The psychometric analyses are presented in Table 4. For mothers and fathers combined, the sensitivity for each risk factor was between 70% and 100%, with the exception of IPV, where it was 47%. Specificity was highest for IPV and lowest for depressive symptoms. NPVs were high and PPVs were low to moderate for all risk factors.

For depressive symptoms and IPV, sensitivity was significantly higher for mothers compared to fathers. This difference was particularly evident for IPV, where only 27% of fathers with this problem on the CAS were detected by the PSQ-S compared to 52% of mothers. Significant differences between genders were found for NPV values regarding depressive symptoms and for NPV and PPV regarding alcohol misuse.

**Table 4:** Sensitivity, specificity, positive (PPV) and negative (NPV) predictive values for PSQ-S risk factors in relation to the standard instruments*

	Sensitivity (%)	Specificity (%)	PPV (%)	NPV (%)
Economic worries				
Mothers (n = 486)	83	82	46	96
Fathers (n = 109)	64	87	43	94
Total (n = 595)	80	83	45	96
**Depressive symptoms**				
Mothers (n = 464)	**93**	65	31	**98**
Fathers (n = 104)	**67**	61	18	**93**
Total (n = 568)	89	64	29	97
**Parental stress**				
Mothers (n = 401)	79	76	30	96
Fathers (n = 87)	71	79	23	97
Total (n = 488)	78	76	29	97
**Intimate partner violence**				
Mothers (n = 447)	**52**	91	70	**83**
Fathers (n = 103)	**27**	93	64	**72**
Total (n = 550)	47	91	69	81
**Alcohol misuse**				
Mothers (n = 464)	73	90	**15**	**99**
Fathers (n = 104)	67	93	**46**	**97**
Total (n = 568)	70	91	22	99
**PSQ-S as a whole**				
Mothers (n = 427)	94	51	66	88
Fathers (n = 95)	90	56	69	83
Total (n = 522)	93	52	67	87

* Numbers in bold denote significant differences between genders using Perason’s chi-square (*p* < 0.05)

Analyses of internal consistency showed Cronbach’s alpha values of 0.31 for financial worries, 0.66 for depressive symptoms, 0.50 for parental stress, 0.69 for IPV, 0.31 for alcohol misuse and 0.58 for the psychosocial composite of the PSQ-S.

Exploratory factor analysis for the 14 PSQ-S items regarding psychosocial risk factors gave rise to five components (Table 5). There was considerable overlap between some of the targeted domains. In component 1, items regarding concerns about being able to afford monthly expenses, often feeling extremely stressed, feeling that the child was especially difficult and needing more help with the child factored together with feeling depressed and feeling less joy and interest (component 1). In component 3, not being able to afford food or clothes for the child factored together with questions regarding child difficultness, needing more help with the child and being afraid of losing control towards the child. In component 5, the item regarding frequency of alcohol consumption factored together with the question about child difficultness. The three items regarding IPV clearly constituted a separate component (component 2) as did the items regarding the number of alcoholic drinks consumed per day and the frequency of binge drinking (component 4).

**Table 5:** Rotated component matrix from exploratory factor analysis using varimax rotation showing component makeup and factor loadings for items of the PSQ-S regarding psychosocial risk factors

	Component				
Item	1	2	3	4	5
During the past year, have you been worried that your money will not last for the whole month?	0.586	0.017	0.029	0.221	− 0.341
During the past year, have you not been able to afford to buy food or clothes that the child needs?	0.084	− 0.028	0.645	0.137	− 0.197
Have you felt down, depressed or had feelings of hopelessness in recent months?	0.826	0.002	0.022	0.014	0.036
In recent months, have you felt less interest in or enjoyment about things that you otherwise usually enjoy or are interested in?	0.762	0.002	0.104	0.016	0.160
Do you often feel extremely stressed?	0.565	0.176	0.148	0.085	− 0.156
Do you feel that your child is particularly difficult to handle?	0.387	0.171	0.429	− 0.072	0.418
Do you need more help with your child?	0.385	0.077	0.512	− 0.168	0.221
Are you worried that you may lose control towards your child?	0.005	− 0.096	0.745	0.048	− 0.036
Has your current or a former partner ever put you down, insulted or exercised control over you, for example decided who you can meet, how much money you can have, which clothes you are allowed to wear?	0.132	0.712	− 0.069	0.132	0.004
Has your current or a former partner ever threatened, pushed, hit, kicked or subjected you to any other type of bodily harm?	0.010	0.820	0.024	0.018	− 0.020
Have you ever been afraid of your partner or another person in your close circle?	0.019	0.773	− 0.017	− 0.073	− 0.089
How often do you have a drink containing alcohol?	− 0.083	− 0.137	− 0.130	0.067	0.778
How many drinks containing alcohol (see example below) do you have on a typical day when you are drinking?	0.049	0.081	0.149	0.809	− 0.220
How often do you have six or more drinks on one occasion?	0.123	− 0.003	− 0.042	0.830	0.273

## Discussion

The present study is the first analysis of the Swedish version of the SEEK-PSQ and its psychometric properties. As a whole, the PSQ-S showed good sensitivity and NPV values and low to moderate specificity and PPV values compared to the standardized instruments. The psychometric properties varied considerably between domains, with sensitivity ≥ 80% for economic worries and depressive symptoms, 78% for parental stress, 47% for IPV and 70% for alcohol misuse. Sensitivity and NPV were significantly higher for women compared to men with regard to depressive symptoms and IPV. Internal consistency was low to moderate for the separate domains and moderate for the PSQ-S composite. Factor analysis corroborated the domains of IPV and alcohol misuse as separate components, while items from the remaining domains were intermixed in three components, suggesting that the targeted domains of economic worries, depressive symptoms and parental stress are not represented as separate and unique factors in the PSQ-S.

In the areas that can be compared, the performance of the PSQ-S was similar to or better than the original U.S. version of the PSQ. Analyses of the U.S. version showed that, for food insecurity, sensitivity was 59%, specificity was 87%, PPV was 70% and NPV was 81% [26]. For depression, sensitivity was 74%, specificity 80%, PPV 36%, and NPV 95%, for IPV sensitivity was 29%, specificity 92%, PPV 41%, and NPV 88%, and for alcohol abuse, sensitivity was 13%, specificity 96%, PPV 33%, and NPV 87% [23–25]. These comparisons deserve a note of caution, as the standardized instruments and methodologies used for validation differ from the present analyses.

Over two thirds of parents had a positive screen for at least one risk factor in the PSQ-S. Although this proportion seems strikingly high, other studies focusing on specific psychosocial issues among parents have shown comparable results. Prevalence studies in Sweden have reported that 23% of children 0–4 years of age live in families with low economic standard, 23% of infants have at least one parent with depressive symptoms, 20% of children live with a parent who drinks too much and 14% have witnessed IPV between their parents [44–47]. Studies from the US have shown similar prevalence rates for these problems, and around 13% of children live in families where at least one parent has a high level of stress [48]. Our results suggest that parents of young children disclosed these problems to a great extent in the PSQ-S in the present setting.

High composite sensitivity of the PSQ-S indicates that few parents who have problems are missed and high NPV suggests that most parents with a negative screen on the PSQ-S do not have the problem. Both of these aspects are important when assessing the psychosocial environment in families with young children. Specificity and PPVs however were low to moderate for the PSQ-S as a whole, indicating a relatively large number of false positives. This may be acceptable in the context of the SEEK model, as a positive screen should quickly be followed by a brief assessment clarifying the parent’s situation and should not entail a burden to healthcare workers, unwarranted referrals or parental concern.

Although sensitivity and NPV were high in total for economic worries, differences were seen between genders particularly regarding sensitivity. The lower sensitivity for fathers should be interpreted with caution due to the small number who participated. To our knowledge, research regarding screening instruments for financial problems is very limited [49]. The questions used for comparison in our analyses are regularly administered by the Public Health Agency of Sweden to assess financial vulnerability and associations with public health outcomes, although their psychometric properties have not been reported.

Previous research has indicated that screening instruments for depression often are more effective at identifying this problem in women than men [50]. This is in line with our findings on the PQS-S. The reasons for gender differences in self-reports for this risk factor are likely complex, deriving in part from differences in how women and men identify and label emotionally or culturally charged experiences [29, 30, 50]. Again, the relatively small number of fathers warrants caution in this interpretation. We chose the lower cutoff level of 7 points for the HADS, which signals possible depression. This is a clinically relevant level of concern from a preventive health service perspective. When the higher cutoff level of 11 points was used, indicating probable depression requiring medical evaluation, sensitivity and NPV rose to 100% for both women and men, while specificity decreased to about 58% (data not shown).

Regarding parental stress, the cutoff level (90th percentile) for the reference instrument (SPSQ) yielded moderate sensitivity and specificity for the PSQ-S. Approximately one fifth of parents in the top 10% of SPSQ scores were missed, and, one fifth of those under this cutoff had a positive screen in the PSQ-S. As mentioned above, false positive screens are not necessarily problematic in the context of CHC visits, as the most common outcome is a discussion with the nurse during the visit. We could find no previous studies regarding screening instruments for parental stress that provided the psychometric properties presented here, therefore comparisons can not be made. The high NPV for parental stress indicates that most parents with a negative screen on the PSQ-S do not have a high score on the SPSQ.

IPV stands out as the domain with the lowest sensitivity for both mothers and fathers. In general, sensitivity has been found to be low for survey instruments about IPV with considerable variation between methods [51]. This may in part be due to the potentially sensitive nature of the issue, where feelings of shame, guilt or fear or retaliation from a violent partner may make it difficult to answer truthfully [52]. In addition, parents may not be inclined to disclose IPV if they are afraid that it may lead to a report to child protective services or the police. In Sweden, exposing children to parental IPV is a criminal offense, which may also deter some parents from disclosing their own IPV exposure. Questionnairis using several detailed questions about specific acts of violence have been shown to capture experiences of violence more effectively than singular or more general questions [53]. As the PSQ-S uses only three items to assess IVP exposure, it might therefore be expected to show low sensitivity compared to the CAS. The difference in sensitivity between genders indicates that fathers who experienced IPV were not readily identified using the PSQ-S. Little is known about screening for IPV among men, and more research is needed in this regard [54].

In the present study, the specificity for IPV was over 90%, indicating that only a few who had not experienced IPV were falsely identified as having been exposed. The NPV for IPV was also the lowest among the risk factors, indicating that as many as a fourth of fathers and a fifth of mothers with a negative screen were likely exposed and missed being identified. This may be seen as a shortcoming of the PSQ-S.

An important component of the SEEK model is that parents answer the PSQ-S several times during the child’s first five years of life, which offers many opportunities to reflect upon and respond to the questions. Seeds may be planted that the nurse cares about them and also about this problem. Thus parents may later disclose IPV as well as other problems, when they may be ready to address their situation. For this reason, missing the earlier identification may not be so consequential. Another consideration is that without systematic screening, many instances of IPV are likely missed. Given the importance of this problem, the modest sensitivity may be acceptable albeit suboptimal. Further research is needed to identify questions with greater sensitivity.

Sensitivity regarding alcohol misuse was lower than expected, given that the AUDIT-C, which is included in the PSQ-S, has previously been shown to be valid in primary care screening compared to the full version of AUDIT [55]. The cutoff level (4 points for women and 5 for men) applied in the current study was higher compared to the original cutoff (3 points for women and 4 points for men) used in previous validation studies from the United States. The higher cutoff has been shown to have optimal psychometric properties in European settings and is commonly used in clinical practice in several countries, including Sweden, to identify significant alcohol misuse and to avoid overidentification [56]. Had we used the lower cutoff, sensitivity would have risen to over 90% while specificity would have fallen to 70%. In a previous study, we found that CHS nurses felt that discussions with parents regarding alcohol consumption were the most challenging among the PSQ-S domains, often evoking pushback from parents [57]. This suggests that costs in terms of time to discuss many false positives and possible parental irritation should be weighed against potential health benefits for a small number of parents with scores near the lower threshold for alcohol misuse.

### Methodoligical considerations

Strengths of the study include recruitment of a relatively large sample of both mothers and fathers from the CHCs at which the PSQ-S is intended to be used. This adds to the ecological validity of the instrument in the Swedish CHS setting and the knowledge base particularly regarding fathers, which is inadequate at present. The methodical adaptation process involved in developing the Swedish version of the PSQ may also be seen as conducive to its applicability in clinical pratice. This should be further evaluated within the framework of the randomized trial.

The standardized instruments for comparison were mostly ones considered optimal albeit less than “gold standards”. In addition, logistical and cost constraints precluded a thorough clinical evaluation for all parents in the study. There is a risk that the standard instruments used here did not accurately identify the phenomena they were intended to measure [58] or that they measured problems that did not quite match the screening questions. Previous studies however have shown their validity [32, 35, 37, 39, 40].

The sample in the present study differed in several ways compared to the population in general. The extent to which this may have influenced the results cannot be evaluated, but as the sample is not representive with regard to gender, educational level or country of birth, generalisability of the results may have been affected.

For all the risk factors, the proportion of parents with a positive screen was higher in the study sample compared to parents who completed the PSQ-S at child health visits during the first months of the intervention in Dalarna county (financial worries 21%, depressive symptoms 33%, parental stress 20%, alcohol misuse 5%, IPV 11%, any positive screen 58%). This may relate to skewing due to self-selection, e.g. those who chose to participte may have experienced psychosocial problems in the past or at the time of the study, or they may represent a group that is more inclined to disclose such problems. However, the differences in rates could also be an influence of the setting. Results from a previous national survey in Sweden using the same questions regarding IPV showed prevalence rates similar to those found here, which suggests that the context in which the questions are asked may affect the respondents’ willingness to disclose psychosocial problems [59].

## Conclusions

As a whole, the PSQ-S performed well, with high sensitivity (93%) and NPV (87%), indicating that most parents with and without the targeted psychosocial risk factors were correctly identified. The psychometrics were good for identification of economic worries, depressive symptoms, and parental stress and adequate for alcohol misuse, but were poorer for IPV. The problems were commonly reported among both mothers and fathers, and few gender differences in the psychometric properties of the PSQ-S were identified. Although further development may be necessary to improve sensitivity for identification of IPV, the results suggest that the PSQ-S may be a valuable tool to identify the targeted psychosocial risk factors it is intended to among both mothers and fathers with young children in the CHS setting.

### Electronic supplementary material

Below is the link to the electronic supplementary material.

Supplementary Material 1

## Data Availability

All summarized data can be made available for use by other researchers following review. For information, contact the corresponding author (Maria Engström maria.engstrom@kbh.uu.se ).

## Abbreviations

AUDIT: Alcohol Use Disorders Identification Test
CAS: Composite Abuse Scale
CHC: Child Health Center
CHS: Child Health Services
CM: Child maltreatment
HADS: Hospital Anxiety and Depression Scale
IPV: Intimate Partner Violence
MI: Motivational Interviewing
PSQ: Parent Screening Questionnaire
PSQ-S: Parent Screening Questionnaire - Swedish Version
SEEK: The Safe Environment for Every Kid model
SPSQ: Swedish Parenthood Stress Questionnaire
