# Supplementary material for: Validation of the Swedish version of the safe environment for every kid (SEEK) parent screening questionnaire
Source: BMC Public Health. 2023 Oct 12;23:1989. doi: 10.1186/s12889-023-16792-4 (PMC10571478; doi:10.1186/s12889-023-16792-4)
Supplement: Supplementary file 1 — Supplementary Material 1 [file 12889_2023_16792_MOESM1_ESM.pdf]

The SEEK-PSQ-S

SEEK™ Safe Environment for Every Kid

(D)

Dear parent or guardian,

Being a parent is not always easy. One of our most important tasks at the child health centre (BVC) is to help families so that children have a safe home environment. For this reason, we offer all the families who visit us to answer the questions below. The questions are about things that can affect many families. The questions concern you and the child visiting the health centre today. Filling in the form is voluntary, and you can choose to answer all, some or none of the questions.

Child's gender:__________ Child's age: ____years _______ months   Parent's gender: _________

- ☐ Yes ☐ No Do you know what number to call if your child has swallowed something poisonous?
- ☐ Yes ☐ No Do you have smoke alarms installed in your home?
- ☐ Yes ☐ No Does anyone who lives in your home smoke?
- ☐ Yes ☐ No During the past year, have you been worried that your money will not last for the whole month?
- ☐ Yes ☐ No During the past year, have you not been able to afford to buy food or clothes that the child needs?
- ☐ Yes ☐ No Have you felt down, depressed or had feelings of hopelessness in recent months?
- ☐ Yes ☐ No In recent months, have you felt less interest in or enjoyment about things that you otherwise usually enjoy or are interested in?
- ☐ Yes ☐ No Do you often feel extremely stressed?
- ☐ Yes ☐ No Do you feel that your child is particularly difficult to handle?
- ☐ Yes ☐ No Do you need more help with your child?
- ☐ Yes ☐ No Are you worried that you may lose control towards your child?
- ☐ Yes ☐ No Has your current or a former partner ever put you down, insulted or exercised control over you, for example decided who you can meet, how much money you can have, which clothes you are allowed to wear?
- ☐ Yes ☐ No Has your current or a former partner ever threatened, pushed, hit, kicked or subjected you to any other type of bodily harm?
- ☐ Yes ☐ No Have you ever been afraid of your partner or another person in your close circle?

How often do you have a drink containing alcohol?

☐ Never ☐ Monthly or less ☐ 2-4 times a month ☐ 2-3 times a week ☐ 4 or more times a week

How many drinks containing alcohol (see example below) do you have on a typical day when you are drinking?

☐ 1-2 ☐ 3-4 ☐ 5-6 ☐ 7-9 ☐ 10 or more

How often do you have six or more drinks on one occasion?

☐ Never ☐ Less than monthly ☐ Monthly ☐ Weekly ☐ Daily or almost daily

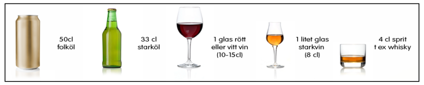

☐ Yes ☐ No Are there any other problems for which you would like to receive help today?

Many Thanks!

English

US version of the PSQ

SEEK™ Safe Environment for Every Kid

Parent Questionnaire (PQ)

Dear Parent or Caregiver: Being a parent is not always easy. We want to help families have a safe environment for kids. So, we're asking everyone these questions. They are about problems that affect many families. If there's a problem, we'll try to help.

Please answer the questions about your child being seen today for a checkup. If there's more than one child, please answer "yes" if it applies to any one of them. This is voluntary. You don't have to answer any question you prefer not to.

Today's Date: ___/___/___   Child's Name: ___________________________

Child's Date of Birth: ___/___/___

PLEASE CHECK

- ☐ Yes ☐ No Do you need the phone number for Poison Control?
- ☐ Yes ☐ No Do you need a smoke detector for your home?
- ☐ Yes ☐ No Does anyone smoke tobacco at home?
- ☐ Yes ☐ No In the last year, did you worry that your food would run out before you got money or Food Stamps to buy more?
- ☐ Yes ☐ No In the last year, did the food you bought just not last and you didn't have money to get more?
- ☐ Yes ☐ No Do you often feel your child is difficult to take care of?
- ☐ Yes ☐ No Do you sometimes find you need to hit/spank your child?
- ☐ Yes ☐ No Do you wish you had more help with your child?
- ☐ Yes ☐ No Do you often feel under extreme stress?
- ☐ Yes ☐ No In the past month, have you often felt down, depressed, or hopeless?
- ☐ Yes ☐ No In the past month, have you felt very little interest or pleasure in things you used to enjoy?
- ☐ Yes ☐ No In the past year, have you been afraid of your partner?
- ☐ Yes ☐ No In the past year, have you had a problem with drugs or alcohol?
- ☐ Yes ☐ No In the past year, have you felt the need to cut back on drinking or drug use?
- ☐ Yes ☐ No Are there any other problems you'd like help with today?

Please give this form to the doctor or nurse you're seeing today. Thank you!

©2012, University of Maryland School of Medicine
